# Colorectal Cancer in Individuals with Cirrhosis: A Population-Based Study Assessing Practice Patterns, Outcomes, and Predictors of Survival

**DOI:** 10.3390/curroncol30110690

**Published:** 2023-10-30

**Authors:** Sunil Patel, Kelly Brennan, Lisa Zhang, Maya Djerboua, Sulaiman Nanji, Shaila Merchant, Jennifer Flemming

**Affiliations:** 1 Department of Surgery, Queen’s University, Kingston, ON K7L 2V7, Canada; 2 Cancer Care and Epidemiology, Queens Cancer Research Institute, Kingston, ON K7L 3N6, Canada; 3 Department of Surgery, Ottawa University, Ottawa, ON K1H 8L6, Canada; 4 ICES Queens, Kingston, ON K7L 3L4, Canada; 5 Department of Medicine, Queen’s University, Kingston, ON K7L 3N6, Canada

**Keywords:** cirrhosis, colorectal cancer, cancer treatment, survival, mortality

## Abstract

Those with cirrhosis who develop colorectal cancer (CRC) are an understudied group who may tolerate treatments poorly and are at risk of worse outcomes. This is a retrospective cohort study of 842 individuals from Ontario, Canada, with a pre-existing diagnosis of cirrhosis who underwent surgery for CRC between 2009 and 2017. Practice patterns, overall survival, and short-term morbidity and mortality were assessed. The most common cirrhosis etiology was non-alcoholic fatty liver disease (NAFLD) (52%) and alcohol-associated liver disease (29%). The model for end-stage liver disease score (MELD-Na) was available in 42% (median score of 9, IQR7-11). Preoperative radiation was used in 62% of Stage II/III rectal cancer patients, while postoperative chemotherapy was used in 42% of Stage III colon cancer patients and 38% of Stage II/III rectal cancer patients. Ninety-day mortality following surgery was 12%. Five-year overall survival was 53% (by Stages I–IV, 66%, 55%, 50%, and 11%, respectively). Those with alcohol-associated cirrhosis (HR 1.8, 95% CI 1.5–2.2) had lower survival than those with NAFLD. Those with a MELD-Na of 10+ did worse than those with a lower MELD-Na score (HR 1.9, 95% CI 1.4–2.6). This study reports poor survival in those with cirrhosis who undergo treatment for CRC. Caution should be taken when considering aggressive treatment.

## 1. Introduction

Both liver cirrhosis and colorectal cancer are expected to rise over the next several decades, especially among young adults [1,2,3]. Those with cirrhosis represent a challenging population for the management of cancer for several reasons. Firstly, the development of portal hypertension in those with cirrhosis can complicate surgical decision-making as it is associated with thrombocytopenia, an increased risk of peri-operative bleeding, and an increased risk of postoperative hepatic decompensation. Further, the use of certain chemotherapeutic regimens is contraindicated in the setting of altered liver function. Finally, most patients with cirrhosis are not included in cancer trials, resulting in a lack of evidence to help delineate the risks and benefits of cancer treatments in the context of underlying cirrhosis. 

Treatment of colorectal cancer in those with an existing diagnosis of cirrhosis can be a challenge. Curative intent treatment typically requires major surgical resection and may include liver-toxic chemotherapy [4,5]. Previous studies have demonstrated that individuals with cirrhosis experience high risks of perioperative mortality (up to 30%) and major morbidity (between 40 and 70%) [6,7,8,9,10,11,12,13]. Chemotherapy may be risky, and it has been found to be associated with liver injury in some studies [14,15]. Conversely, there is the potential for harm resulting from the underuse of curative intent treatment in individuals with cirrhosis [16]. Due to a reluctance to offer chemotherapy as well as limits to the extent of surgical resection in those with pre-existing liver cirrhosis, some studies have found an increased risk of metastasis following colorectal cancer surgery compared to those without cirrhosis [17,18].

Despite the increasing incidence of both cirrhosis and colorectal cancer, there have been few contemporary studies assessing outcomes among this complex and unique group. Thus, the objectives of this study were to (1) determine the proportion of patients who receive guideline-recommended cancer treatments (such as radiation and chemotherapy); (2) determine the overall survival; (3) determine the associations between cirrhosis etiology and/or severity and survival among a cohort of patients with cirrhosis and CRC.

## 2. Methods

This is a retrospective population-level study of all individuals with cirrhosis who underwent surgery for colorectal cancer in Ontario, Canada, from January 2009 to December 2017 with outcome events followed until the end of 2020. This study conforms to the Strengthening the Reporting of Observational Studies in Epidemiology (STROBE) guidelines [19].

### 2.1. Data Sources

This study uses routinely collected healthcare datasets stored at ICES. These datasets include health administrative data that are routinely generated upon the delivery of a healthcare service to an individual, as well as demographic and census data. The datasets were linked at the individual level using a unique encrypted identifier and analyzed at ICES (Table S1).

### 2.2. Participants

Individuals with incident or prevalent cirrhosis were identified using a previously validated algorithm [20]. The algorithm included a combination of physician visits and hospital diagnosis codes. Within this group, those with an incident diagnosis of colorectal cancer from 2009 to 2017 in the Ontario Cancer Registry (OCR) who underwent surgery were included. The OCR contains a near-complete capture of incident colorectal cancers, with >95% being microscopically confirmed [21,22]. Surgery was identified using physician billing codes from the OHIP physician claims database (Table S2). We excluded individuals with the following criteria: diagnosis of cirrhosis after diagnosis of colorectal cancer; did not have a valid identifier; were not linkable to other ICES datasets; underwent liver transplantation prior to surgery; no corresponding hospital record for their surgical admission; and under 18 years of age at the time of surgery. 

### 2.3. Variables

Patient demographics were derived and included age at the time of surgery, sex, and socioeconomic status. Co-morbid illness was defined using the Charlson co-morbidity index. Specific pre-existing comorbidities were identified in patients based on ICES-validated definitions [23,24,25,26,27]. The etiology of cirrhosis was derived from a validated hierarchical algorithm previously described [28] and included viral hepatitis, autoimmune/other, alcohol-related, and NAFLD. Viral hepatitis and autoimmune/other were grouped together as “other” in our analyses. The severity of cirrhosis indicators included models for end-stage liver disease (MELD-Na) and a history of liver decompensation. MELD-Na was calculated from the most recent lab results available in the OLIS within one year of surgery. The history of liver decompensation was based on a hospital diagnosis in CIHI DAD for variceal bleeding, ascites, hepatic failure, or hepatorenal syndrome within 2 years prior to surgery. Cancer characteristics were derived from OCR, including anatomic location (colon and rectosigmoid vs. rectal) and stage of cancer (Stages I–IV). 

Surgery type was categorized as resection with anastomosis, resection without anastomosis (permanent ostomy), or resection with anastomosis and proximal diversion based on the fee code that was billed in OHIP at the time of surgery (Table S3). Surgical acuity was based on admission status (emergency or elective) in the corresponding CIHI DAD hospital record. Medical or radiation oncology assessments were also based on physician claims in OHIP with service dates between 6 months prior to surgery and 6 months after surgery (see Table S3). Radiation therapy courses were identified from the ALR Radiation Planning and Treatment Activity data and based on treatment records. Receipt of chemotherapy was obtained from ALR Systemic Drug Delivery data and based on treatment records. Both radiation and chemotherapy were classified as pre-surgery (<6 months prior) or post-surgery (<6 months afterwards). 

The primary outcomes were 5-year overall survival outcome and overall survival as a time-to-event outcome. Secondary outcomes included the receipt of guideline-recommended neoadjuvant and adjuvant therapies as described in well-established clinical practice guidelines [4,5].

Other short-term outcomes included in-hospital mortality, 90-day mortality, intensive care unit (ICU) admission, hospital and ICU length of stay, 90-day readmission and 90-day emergency department (ED) visit, and hepatic decompensation [20]. These data were derived from the RPDB or CIHI-DAD.

### 2.4. Statistical Analyses

Counts and proportions were calculated for categorical variables and means with standard deviations or medians with interquartile ranges for continuous variables. We used chi-square statistical tests to compare categorical proportions, one-way analysis of variance to compare means, and Kruskal–Wallis test as a non-parametric approach to compare median values between cancer-type groups. For cells with small numbers (i.e., <6 individuals) ranges are presented to protect the anonymity of the patient population, as required by ICES.

Postoperative outcomes were described according to cancer type along with counts and proportions for categorical outcomes and means with standard deviations or medians with interquartile ranges for continuous outcomes. In-hospital and 90-day mortality was further elucidated by the MELD-Na score. Five-year survival was evaluated using Kaplan–Meier curves and stratified according to cancer type and stage. Predictors associated with postoperative mortality during follow-up were assessed using Cox proportional hazards models. Patients were followed from the time of surgery until death or 31 December 2020. Predictors were selected a priori and evaluated for violation of the proportional hazards assumption using Schoenfeld residuals. We performed a sensitivity analysis by repeating the multivariable Cox models for predictors of postoperative mortality in a subset of the cohort that had an available MELD-Na score and included MELD-Na as a predictor of interest. We also performed a sensitivity analysis, excluding those who died within 90 days of surgery.

Results were considered significant at *p*-value < 0.05. All data were prepared and analyzed using the SAS Enterprise Guide, Version 7.1 (SAS Institute Inc., Cary, NC, USA). 

## 3. Results

**Figure 1:**
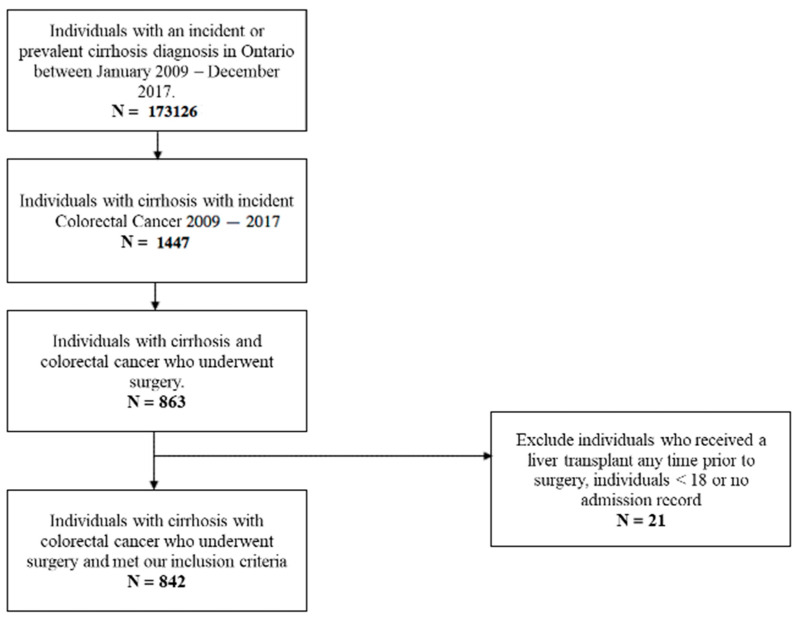
Flowsheet of included patients.

A total of 842 individuals with cirrhosis underwent surgery for colorectal cancer, including 696 individuals with colon cancer and 146 with rectal cancer (Figure 1). Demographics, comorbidities, cirrhosis etiology and severity, cancer stage, and surgical intervention are presented in Table 1. The most common cirrhosis etiology was NAFLD (*n* = 441, 52%), followed by ALD (*n* = 243, 29%), viral hepatitis (*n* = 98, 11.6%), and autoimmune or other (*n* = 60, 7.1%). A small number had a history of hepatic decompensation (*n* = 29–33, 3–4%). The MELD-Na was available for 353 individuals (42%), with a median score of 9 (IQR 7–11). Cancer stages included Stage I disease (*n* = 215, 25.5%), Stage II disease (*n* = 274, 32.5%), Stage III disease (*n* = 261, 31%), and Stage IV disease (*n* = 60–64, 7.1–7.6%). Most individuals underwent elective surgery (*n* = 674, 80.1%). In those with colon cancer (*N* = 696), the most common procedure was resection with anastomosis (*n* = 512, 73.6%), followed by resection and permanent ostomy (*n* = 153, 22.0%). Few individuals with colon cancer underwent anastomosis and proximal diversion (*n* = 31, 4.5%). Those with rectal cancer (*N* = 146) most commonly had a resection with a permanent ostomy (*n* = 84, 57.5%) or resection with anastomosis and proximal diversion (*n* = 49, 33.6%). Few individuals underwent resection and anastomosis without diversion (*n* = 13, 8.9%).

### 3.1. Neoadjuvant and Adjuvant Treatment

In those with Stage III colon cancer, approximately half (119/213, 56%) had a medical oncology assessment, with 42% (90/213) ultimately receiving adjuvant chemotherapy. In those with locally advanced rectal cancer (i.e., Stage II/III), 70% (62/89) had a radiation oncology assessment, with 62% (55/89) receiving preoperative radiation. Similar to Stage III colon cancer, about half (46/89, 52%) of those with locally advanced disease had a medical oncology assessment and 38% (34/89) ultimately received adjuvant chemotherapy. 

### 3.2. Short-Term Outcomes

Short-term outcomes are presented in Table 2. The in-hospital and 90-day mortality outcomes were 7.2% and 12.1%, respectively. Ninety-day postoperative hepatic decompensation occurred in 9%. In those who survived to discharge, 90-day readmission (27%) and emergency department visits (40%) were common (Table 2). The majority of those who underwent surgery with an available MELD-Na score had a score < 10 (*n* = 219/353, 62%), with few having a score >20 (*n* = 10/353, 3%). The 90-day mortality in those with a MELD-Na <10 was 6.8% vs. 22% in those with a MELD-Na >10 (*p* < 0.001) (Table 2).

### 3.3. Overall Survival

**Figure 2:**
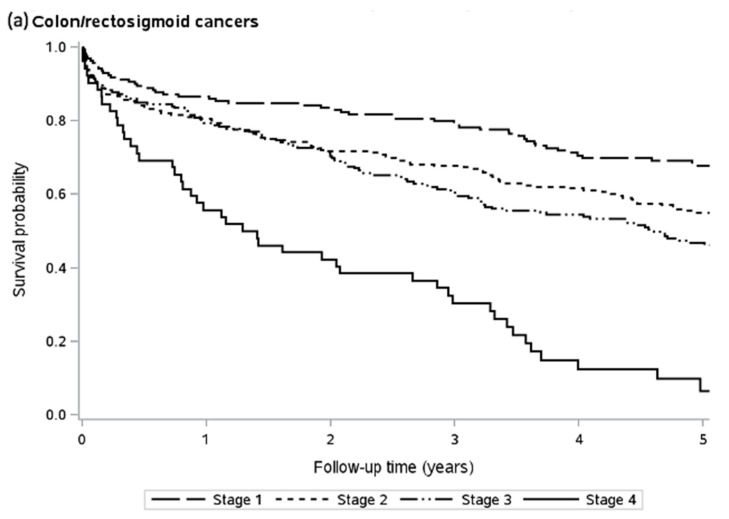

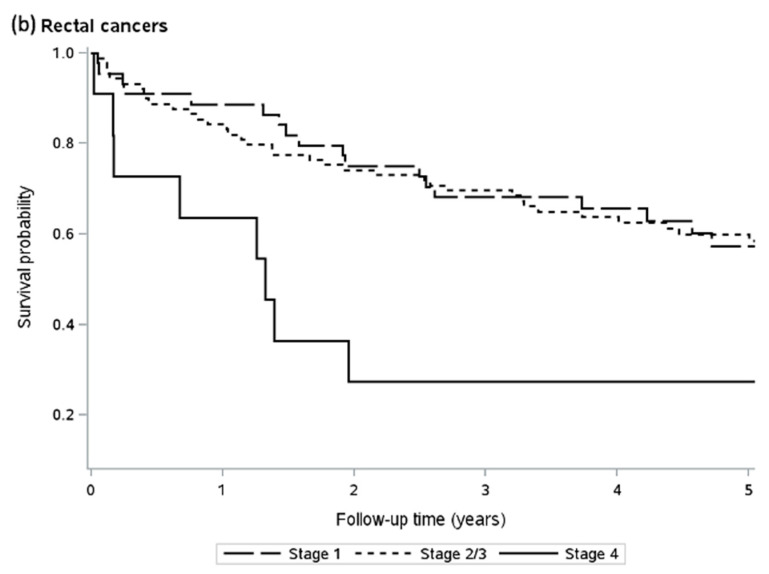
Kaplan–Meier survival estimates according to cancer region and stage.

The 5-year overall survival for the cohort was 53%. Table 2 reports the survival according to cancer type, while Figure 2 presents the Kaplan–Meier survival curve. Interestingly, those with locally advanced rectal cancer (Stage II/III) had a similar 5-year survival to those with Stage I rectal cancer (Stage I 57.2% vs. Stage II/III 59.9%). Those with Stage IV disease did very poorly, with a 5-year overall survival of just 11.3%. After excluding those who died within 90 days of surgery, the 5-year overall survival was 60% for all stages. 

Cox proportional hazards regression was completed for all individuals in the cohort (Table 3) and was stratified according to colon vs. rectal cancer subtype (Table 4). Those with ALD cirrhosis and those with “other” etiologies (viral hepatitis, autoimmune, and other) of cirrhosis did worse than those with NAFLD (alcohol-related HR 1.80, 95% CI 1.45–2.23; other HR 1.48, 95% CI 1.13–1.93; NAFLD [ref]). Other risk factors associated with worse survival included increasing age, increasing Charlson comorbidity, and income quintile. As expected, the stage of disease was also associated with worse survival for the cohort. The colorectal cancer subtype (colon vs. rectum) did not demonstrate an association (rectal HR 0.97, 95% CI 0.75–1.25; colon [ref]). Stratified analysis based on colon vs. rectum demonstrated similar findings to the overall cohort, except for the stage of disease in rectal cancer patients. This analysis demonstrated only Stage IV to be associated with worse survival (Table 4). Sensitivity analysis, in which those who died within 90 days were excluded, demonstrated similar associations (data not shown).

Cox proportional hazards regression was also completed for the subgroup with MELD-Na score available at the time of surgery (Table 3). Those with a MELD-Na score of >10 had a significantly higher hazard of mortality compared to those with a MELD-Na score ≤ 10 (HR 1.88, 95% CI 1.37–2.57).

## 4. Discussion

The present study reports practice patterns and outcomes in those with cirrhosis who develop colorectal cancer and undergo surgical resection. The 5-year overall survival for the cohort was 53%. Cirrhosis etiology was associated with overall survival, while MELD-Na score was predictive of increased 90-day mortality. A number of other major short-term complications were common, including 90-day mortality (12%).

### 4.1. Meaning of the Study

The present study is the largest and most contemporary report of outcomes in those with cirrhosis who develop colorectal cancer and receive surgery. Several older studies have reported long-term survival [29,30,31,32,33]. These studies included between 40 and 453 individuals who were diagnosed with colorectal cancer between 1976 and 2014. The characteristics of the present cohort differ from these older studies in that our data are more comprehensive and are more representative of the current heterogeneous population with cirrhosis. For instance, in the studies by Lee et al. [31] and Han et al. [30], >70% of the cohort had viral hepatitis, while the study by Sabbagh et al. [32] and Gervaz et al. [29] found ALD to be the most common etiology of cirrhosis. Furthermore, the largest previous study [33] did not report cancer stage, cirrhosis etiology, or cirrhosis severity. Nevertheless, our reported 5-year survival of 53% is similar to the 55–62% survival reported in more recent studies [31,32,33] and is much improved compared with the 25–35% survival reported in much older studies [29,30]. We also show that a higher MELD score is associated with worse survival. 

An interesting finding from this work is the differences in outcomes based on the etiology of cirrhosis. Those with ALD had a worse survival compared with those with NAFLD (HR 2.21, 95% CI 1.2–4.0, *p* = 0.082). This association persisted even after adjusting for MELD-Na in those with a score available. The reasons for this are not immediately clear from the data but could relate to individuals with ALD having a more advanced liver dysfunction that is not able to be captured using MELD alone such as the degree of ascites and hepatic encephalopathy. Further, individuals with ALD cirrhosis have historically included a higher proportion who are vulnerable due to their social determinants of health, which may influence the ability of patients with ALD to access and engage in care. 

Compared with the general population, the present study demonstrates a lower utilization of adjuvant chemotherapy as well as worse survival in those with cirrhosis. In the general population, approximately 66% of those with Stage III colon cancer and 18% of those with Stage II colon cancer receive adjuvant chemotherapy [34,35]. This is in comparison to our findings of 42% of Stage III and 10% of Stage II receiving adjuvant chemotherapy. 

Interestingly, we found similar survival in those with Stage I vs. Stage II/III disease in those with rectal cancer. This suggests that the etiology and/or severity of liver cirrhosis is likely a more important contributor to long-term survival than the rectal cancer stage. Additionally, a smaller proportion of eligible patients received adjuvant chemotherapy than reported in the general population. This may also contribute to the differences in overall survival, as adjuvant chemotherapy has been associated with improved survival in both colon and rectal cancer patients [36,37]. 

Finally, we demonstrated that those with cirrhosis and colorectal cancer had a worse 5-year survival (53%) compared to that reported for all comers in Canada (65%), based on previous publications [38]. The Canadian survival includes those who did not undergo resection. Our group assessed those with rectal cancer undergoing resection between 2010–2019 and found an overall survival of 78% (vs. 62% in this study) [39].

### 4.2. Strengths and Limitations

This study has several strengths. Due to the relative scarcity of individuals with both a diagnosis of cirrhosis and colorectal cancer, a population-based approach allowed for the inclusion of a large number of individuals. The identification of those with cirrhosis was made using a previously validated algorithm which ensured a low risk of misclassification or inappropriate inclusion. We also identified those with colorectal cancer using the Ontario Cancer Registry, which is highly accurate. For these reasons, there was a low risk of missing individuals with pre-existing cirrhosis who developed colorectal cancer. The type and extent of treatment were determined using well-established sources, while the outcomes were determined using previously validated approaches. As the data for this study were ascertained using linked administrative databases within a single-payer universal healthcare system, loss to follow-up was minimal.

Despite these strengths, several well-described limitations of population-based studies exist within this study [40]. Pertinent to the current study, missing data were an issue. All data elements necessary for MELD-Na score calculation were available in <50% of individuals; however, when sensitivity analysis was completed in those with MELD-Na available, similar associations between risk factors and survival were identified. Another limitation is selection bias, as only those who underwent surgery were included, and we cannot ascertain the factors involved in surgical decision-making. We presume that a number of individuals with cirrhosis were either deemed unfit for curative-intent treatment or declined treatment. Thus, the overall survival we report is likely overestimated. Finally, due to the nature of the study design (retrospective cohort) and completeness of the data sources (i.e., linked administrative databases), there is likely unresolved confounding in our adjusted analysis. Clinical information such as chemotherapy regimen and renal function is not available. 

## 5. Conclusions

Patients with cirrhosis who develop colorectal cancer and undergo surgical resection infrequently receive guideline-recommended treatment, have poor short-term outcomes, and have poor overall survival. Further, compared to the general population, the stage of the disease is less likely to be predictive of long-term survival, especially in those with rectal cancer. We found liver-related factors, including ALD and MELD-Na > 10, to be associated with survival. This information will assist healthcare providers in having detailed discussions with these patients and their caregivers, allowing for enhanced awareness and education pertaining to the anticipated challenges associated with treatment.

## Figures and Tables

**Table 1:** Patient characteristics of those with cirrhosis who underwent surgery for colorectal cancer between 2009 and 2017.

Characteristics	Colon Cancer	Rectal Cancer	Total	*p*-Value
	N = 696	N = 146	N = 842	
Age at Surgery				
<50	23 (3.30)	11 (7.53)	34 (4.04)	0.006
50–59	95 (13.65)	30 (20.55)	125 (14.85)	
60–69	214 (30.75)	44 (30.14)	258 (30.64)	
70–79	228 (32.76)	45 (30.82)	273 (32.42)	
80+	136 (19.54)	16 (10.96)	152 (18.05)	
Sex—n (%)				
Female	287 (41.24)	45 (30.82)	332 (39.43)	0.019
Male	409 (58.76)	101 (69.18)	510 (60.57)	
Income Quintile—n (%)				
1	155 (22.27)	36 (24.66)	191 (22.68)	0.822
2–4	413 (59.34)	84 (57.53)	497 (59.03)	
5	128 (18.39)	26 (17.81)	154 (18.29)	
Charlson Comorbidity index—n (%)				
0	481 (69.11)	94 (64.38)	575 (68.29)	0.39
1–3	141 (20.26)	37 (25.34)	178 (21.14)	
4+	74 (10.63)	15 (10.27)	89 (10.57)	
Cirrhosis Etiology—n (%)				
Viral Hepatitis	81 (11.64)	17 (11.64)	98 (11.64)	0.836
Autoimmune/Other	47 (6.75)	13 (8.90)	60 (7.13)	
Alcohol-related	202 (29.02)	41 (28.08)	243 (28.86)	
NAFLD	366 (52.59)	75 (51.37)	441 (52.38)	
History of hepatic decompensation—n(%)	28 (4.02)	<6	29–33 (3.44–3.92)	0.735
MELD - Na Score				
MELD Score available	290 (41.67)	63 (43.15)	353 (41.92)	
Mean ± SD	9.94 ± 3.97	8.82 ± 3.07	9.74 ± 3.84	0.035
Cancer Stage—n (%)				
Unknown	27 (3.88)	<6	28–32 (3.33–3.80)	0.292
Stage I	171 (24.57)	44 (30.14)	215 (25.53)	
Stage II	233 (33.48)	41 (28.08)	274 (32.54)	
Stage III	213 (30.60)	48 (32.88)	261 (31.00)	
Stage IV	52 (7.47)	8–12 (5.48–8.22)	60–64 (7.13–7.60)	
Type of Surgery—n (%)				
Emergency/Urgent Surgery	159 (22.84)	9 (6.16)	168 (19.95)	<0.001
Resection with anastomosis	512 (73.56)	13 (8.90)	525 (62.35)	<0.001
Resection without anastomosis	153 (21.98)	84 (57.53)	237 (28.15)	
Resection with anastomosis and proximal diversion	31 (4.45)	49 (33.56)	80 (9.50)	

**Table 2:** Short-term outcomes and 5-year overall survival for those with cirrhosis who underwent surgery for colorectal cancer between 2009 and 2017.

Outcomes	Colon Cancer (*N* = 696)	Rectal Cancer (*N* = 146)	Total (*N* = 842)	*p*-Value
In-hospital Mortality—n (%)	55 (7.90)	6 (4.11)	61 (7.24)	0.108
90-day Mortality—n (%)	87 (12.50)	15 (10.27)	102 (12.11)	0.454
Hospital Length of Stay (Mean ± SD)	12.18 ± 14.37	11.58 ± 9.68	12.08 ± 13.67	0.627
Hospital Length of Stay (Median (IQR))	7 (5–13)	9 (6–13)	8 (5–13)	0.007
Any ICU Admission—n (%)	222 (31.90)	44 (30.14)	266 (31.59)	0.678
ICU Length of Stay (Mean ± SD)	6.56 ± 9.42	5.11 ± 7.59	6.32 ± 9.15	0.338
ICU Length of Stay (Median (IQR))	4 (2–7)	4 (2–5)	4 (2–6)	0.596
Hepatic Decompensation (90 Days)—n (%)	69 (9.91)	9 (6.16)	78 (9.26)	0.155
**Alive at Discharge Only**	***N* = 641**	***N* = 140**	***N* = 781**	
Hospital Readmission (90 Days)—n (%)	164 (25.59)	54 (38.57)	218 (27.91)	0.002
Emergency Department Visit (90 Days)—n (%)	247 (38.53)	68 (48.57)	315 (40.33)	0.028
**5-Year Overall Survival (%)**				
All stages	52.09	55.92	52.78	<0.001
Stage I	67.70	57.23	65.55	
Stage II	54.91	59.91 *	54.89	
Stage III	46.91		50.24	
Stage IV	6.63	27.27	11.30	
	**Missing MELD-Na**	**MELD-Na < 10**	**MELD-Na 10+**	
	***N* = 489**	***N* = 219**	***N* = 134**	
In-hospital Mortality—n (%)	35 (7.16)	7 (3.20)	19 (14.18)	<0.001
90-day Mortality—n (%)	57 (11.66)	15 (6.85)	30 (22.39)	

* Stages II and III rectal cancer combined.

**Table 3:** Hazard ratios (HRs) and 95% confidence intervals (CIs) from the Cox proportional hazards model assessing predictors associated with time to mortality.

Characteristic	Full Cohort (*N* = 842)			MELD-Na Available (*N* = 353)		
	HR	95% CI	*p*-Value	HR	95% CI	*p*-Value
Cirrhosis Etiology						
NAFLD	1.000	Ref		1.000	Ref	
Alcohol-related	1.796	1.45–2.23	<0.0001	1.832	1.27–2.64	0.0012
Other	1.481	1.13–1.93	0.0039	1.164	0.78–1.74	0.4546
Age	1.046	1.04–1.06	<0.0001	1.032	1.02–1.05	<0.0001
Sex						
Male	0.983	0.81–1.20	0.8651	0.806	0.59–1.10	0.1750
Female	1.000	Ref		1.000	Ref	
Income Quintile						
1	1.487	1.09–2.02	0.0118	1.696	1.04–2.75	0.0326
2–4	1.246	0.95–1.64	0.1132	1.394	0.91–2.14	0.1277
5	1.000	Ref		1.000	Ref	
Charlson Comorbidity Index						
0	1.000	Ref		1.000	Ref	
1–3	1.726	1.38–2.16	<0.0001	1.466	1.04–2.07	0.0297
4+	3.036	2.32–3.97	<0.0001	3.124	2.09–4.68	<0.0001
Cancer Stage						
Stage I	1.000	Ref		1.000	Ref	
Stage II	1.344	1.03–1.75	0.0273	1.410	0.92–2.16	0.1130
Stage III	1.523	1.17–1.97	0.0015	1.837	1.22–2.78	0.0039
Stage IV	4.415	3.15–6.18	<0.0001	5.173	2.99–8.95	<0.0001
Cancer Region						
Rectal	0.970	0.75–1.25	0.8160	1.157	0.77–1.73	0.4780
Colon	1.000	Ref		1.000	Ref	
MELD-Na score						
10+				1.876	1.37–2.57	<0.0001
<10				1.000	Ref	

**Table 4:** Stratified (colon vs. rectal) hazard ratios (HRs) and 95% CI from the Cox PH model assessing predictors associated with time to mortality.

Characteristic	Colon Cancer (*N* = 696)			Rectal Cancer (*N* = 146)		
	HR	95% CI	*p*-Value	HR	95% CI	*p*-Value
**Cirrhosis Etiology**						
NAFLD	1.000	Ref		1.000	Ref	.
Alcohol-related	1.745	1.38–2.21	<0.0001	2.214	1.23–3.99	0.0082
Other	1.436	1.07–1.93	0.0162	1.769	0.91–3.42	0.0904
**Age**	1.043	1.03–1.06	<0.0001	1.055	1.03–1.08	<0.0001
**Sex**						
Male	0.998	0.81–1.23	0.9857	0.926	0.55–1.57	0.7751
Female	1.000	Ref		1.000	Ref	
**Income Quintile**						
1	1.638	1.17–2.29	0.0041	0.720	0.32–1.62	0.4250
2–4	1.274	0.95–1.72	0.1118	1.001	0.51–1.98	0.9973
5	1.000	Ref	.	1.000	Ref	.
**Charlson Comorbidity Index**						
0	1.000	Ref		1.000	Ref	
1–3	1.927	1.51–2.46	<0.0001	0.956	0.52–1.74	0.8818
4+	3.049	2.27–4.10	<0.0001	3.063	1.47–6.38	0.0028
**Cancer Stage**						
Stage I	1.000	Ref		1.000	Ref	
Stage II	1.391	1.04–1.86	0.0254	1.571	0.79–3.12	0.1966
Stage III	1.675	1.25–2.24	0.0005	1.358	0.73–2.54	0.3369
Stage IV	4.707	3.23–6.86	<0.0001	5.041	2.11–12.05	0.0003

## Data Availability

The dataset from this study is held securely in coded form at ICES. While legal data sharing agreements between ICES and data providers (e.g., healthcare organizations and government) prohibit ICES from making the dataset publicly available, access may be granted to those who meet pre-specified criteria for confidential access, available at www.ices.on.ca/DAS (email: das@ices.on.ca). The full dataset creation plan and underlying analytic code are available from the authors upon request, understanding that the computer programs may rely upon coding templates or macros that are unique to ICES and are therefore either inaccessible or may require modification.

## Supplementary Materials

The following supporting information can be downloaded at: https://www.mdpi.com/article/10.3390/curroncol30110690/s1, Table S1: Data Sources used, with description of data elements; Table S2: Data source and data element codes used for cirrhosis, cancer and surgery variables; Table S3: Ontario Health Insurance Plan (OHIP) fee code used to identify diagnostic imaging and referrals.

## References

[1] Flemming J.A., Dewit Y., Mah J.M., Saperia J., Groome P.A., Booth C.M. (2019). Incidence of cirrhosis in young birth cohorts in Canada from 1997 to 2016: A retrospective population-based study. Lancet Gastroenterol. Hepatol..

[2] Flemming J.A., Djerboua M., Groome P.A., Booth C.M., Terrault N.A. (2021). NAFLD and Alcohol-Associated Liver Disease Will Be Responsible for Almost All New Diagnoses of Cirrhosis in Canada by 2040. Hepatology.

[3] Decker K.M., Lambert P., Bravo J., Demers A., Singh H. (2021). Time Trends in Colorectal Cancer Incidence Rates by Income and Age at Diagnosis in Canada From 1992 to 2016. JAMA Netw. Open.

[4] Benson A.B., Venook A.P., Al-Hawary M.M., Arain M.A., Chen Y.J., Ciombor K.K., Cohen S., Cooper H.S., Deming D., Farkas L. (2021). Colon Cancer, Version 2.2021, NCCN Clinical Practice Guidelines in Oncology. J. Natl. Compr. Cancer Netw. JNCCN.

[5] Benson A.B., Venook A.P., Al-Hawary M.M., Cederquist L., Chen Y.J., Ciombor K.K., Cohen S., Cooper H.S., Deming D., Engstrom P.F. (2018). Rectal Cancer, Version 2.2018, NCCN Clinical Practice Guidelines in Oncology. J. Natl. Compr. Cancer Netw. JNCCN.

[6] Ghaferi A.A., Mathur A.K., Sonnenday C.J., Dimick J.B. (2010). Adverse outcomes in patients with chronic liver disease undergoing colorectal surgery. Ann. Surg..

[7] Käser S.A., Hofmann I., Willi N., Stickel F., Maurer C.A. (2016). Liver Cirrhosis/Severe Fibrosis Is a Risk Factor for Anastomotic Leakage after Colorectal Surgery. Gastroenterol. Res. Pract..

[8] Kazi A., Finco T.B., Zakhary B., Firek M., Gerber A., Brenner M., Coimbra R. (2020). Acute Colonic Diverticulitis and Cirrhosis: Outcomes of Laparoscopic Colectomy Compared with an Open Approach. J. Am. Coll. Surg..

[9] Metcalf A.M., Dozois R.R., Wolff B.G., Beart R.W. (1987). The surgical risk of colectomy in patients with cirrhosis. Dis. Colon. Rectum.

[10] Montomoli J., Erichsen R., Strate L.L., Pedersen L., Nilsson T., Sørensen H.T. (2015). Coexisting liver disease is associated with increased mortality after surgery for diverticular disease. Dig. Dis. Sci..

[11] Nguyen G.C., Correia A.J., Thuluvath P.J. (2009). The impact of cirrhosis and portal hypertension on mortality following colorectal surgery: A nationwide, population-based study. Dis. Colon. Rectum.

[12] Pantel H.J., Stensland K.D., Nelson J., Francone T.D., Roberts P.L., Marcello P.W., Read T., Ricciardi R. (2016). Should We Use the Model for End-Stage Liver Disease (MELD) to Predict Mortality After Colorectal Surgery?. J. Gastrointest. Surg. Off. J. Soc. Surg. Aliment. Tract..

[13] Lacatus M., Costin L., Bodean V., Manuc M., Vasilescu C. (2018). The Outcome of Colorectal Surgery in Cirrhotic Patients: A Case Match Report. Chirurgia.

[14] Robinson S.M., Wilson C.H., Burt A.D., Manas D.M., White S.A. (2012). Chemotherapy-associated liver injury in patients with colorectal liver metastases: A systematic review and meta-analysis. Ann. Surg. Oncol..

[15] Gangi A., Lu S.C. (2020). Chemotherapy-associated liver injury in colorectal cancer. Ther. Adv. Gastroenterol..

[16] Sabbagh C., Cosse C., Chauffert B., Nguyen-Khac E., Joly J.P., Yzet T., Regimbeau J.M. (2015). Management of colon cancer in patients with cirrhosis: A review. Surg. Oncol..

[17] Chiou W.Y., Chang C.M., Tseng K.C., Hung S.K., Lin H.Y., Chen Y.C., Su Y.C., Tseng C.W., Tsai S.J., Lee M.S. (2015). Effect of liver cirrhosis on metastasis in colorectal cancer patients: A nationwide population-based cohort study. Jpn. J. Clin. Oncol..

[18] Monelli F., Besutti G., Djuric O., Bonvicini L., Farì R., Bonfatti S., Ligabue G., Bassi M.C., Damato A., Bonelli C. (2021). The Effect of Diffuse Liver Diseases on the Occurrence of Liver Metastases in Cancer Patients: A Systematic Review and Meta-Analysis. Cancers.

[19] von Elm E., Altman D.G., Egger M., Pocock S.J., Gøtzsche P.C., Vandenbroucke J.P. (2007). Strengthening the Reporting of Observational Studies in Epidemiology (STROBE) statement: Guidelines for reporting observational studies. BMJ (Clin. Res. Ed.).

[20] Lapointe-Shaw L., Georgie F., Carlone D., Cerocchi O., Chung H., Dewit Y., Feld J.J., Holder L., Kwong J.C., Sander B. (2018). Identifying cirrhosis, decompensated cirrhosis and hepatocellular carcinoma in health administrative data: A validation study. PLoS ONE.

[21] Robles S.C., Marrett L.D., Clarke E.A., Risch H.A. (1988). An application of capture-recapture methods to the estimation of completeness of cancer registration. J. Clin. Epidemiol..

[22] (2021). Ontario Cancer Statistics: Data Sources. https://www.cancercareontario.ca/en/statistical-reports/ontario-cancer-statistics-2020/data-sources.

[23] Gershon A.S., Wang C., Guan J., Vasilevska-Ristovska J., Cicutto L., To T. (2009). Identifying patients with physician-diagnosed asthma in health administrative databases. Can. Respir. J..

[24] Lipscombe L.L., Hwee J., Webster L., Shah B.R., Booth G.L., Tu K. (2018). Identifying diabetes cases from administrative data: A population-based validation study. BMC Health Serv. Res..

[25] Schultz S.E., Rothwell D.M., Chen Z., Tu K. (2013). Identifying cases of congestive heart failure from administrative data: A validation study using primary care patient records. Chronic Dis. Inj. Can..

[26] Tu K., Campbell N.R., Chen Z.L., Cauch-Dudek K.J., McAlister F.A. (2007). Accuracy of administrative databases in identifying patients with hypertension. Open Med. Peer-Rev. Indep. Open-Access J..

[27] Gershon A.S., Wang C., Guan J., Vasilevska-Ristovska J., Cicutto L., To T. (2009). Identifying individuals with physcian diagnosed COPD in health administrative databases. COPD.

[28] Philip G., Djerboua M., Carlone D., Flemming J.A. (2020). Validation of a hierarchical algorithm to define chronic liver disease and cirrhosis etiology in administrative healthcare data. PLoS ONE.

[29] Gervaz P., Pak-art R., Nivatvongs S., Wolff B.G., Larson D., Ringel S. (2003). Colorectal adenocarcinoma in cirrhotic patients. J. Am. Coll. Surg..

[30] Han E.C., Ryoo S.B., Park J.W., Yi J.W., Oh H.K., Choe E.K., Ha H.K., Park B.K., Moon S.H., Jeong S.Y. (2017). Oncologic and surgical outcomes in colorectal cancer patients with liver cirrhosis: A propensity-matched study. PLoS ONE.

[31] Lee J.H., Yu C.S., Lee J.L., Kim C.W., Yoon Y.S., Park I.J., Lim S.B., Kim J.C. (2017). Factors affecting the postoperative morbidity and survival of patients with liver cirrhosis following colorectal cancer surgery. Int. J. Color. Dis..

[32] Sabbagh C., Chatelain D., Nguyen-Khac E., Rebibo L., Joly J.P., Regimbeau J.M. (2016). Management of colorectal cancer in patients with cirrhosis: A retrospective, case-matched study of short- and long-term outcomes. Dig. Liver Dis. Off. J. Ital. Soc. Gastroenterol. Ital. Assoc. Study Liver.

[33] Shin N., Han E.C., Won S., Ryoo S.B., Choe E.K., Park B.K., Park K.J. (2020). The prognoses and postoperative outcomes of patients with both colorectal cancer and liver cirrhosis based on a nationwide cohort in Korea. Ann. Surg. Treat. Res..

[34] Booth C.M., Nanji S., Wei X., Peng Y., Biagi J.J., Hanna T.P., Krzyzanowska M.K., Mackillop W.J. (2017). Adjuvant Chemotherapy for Stage II Colon Cancer: Practice Patterns and Effectiveness in the General Population. Clin. Oncol..

[35] Booth C.M., Nanji S., Wei X., Peng Y., Biagi J.J., Hanna T.P., Krzyzanowska M.K., Mackillop W.J. (2016). Use and Effectiveness of Adjuvant Chemotherapy for Stage III Colon Cancer: A Population-Based Study. J. Natl. Compr. Cancer Netw. JNCCN.

[36] Petersen S.H., Harling H., Kirkeby L.T., Wille-Jørgensen P., Mocellin S. (2012). Postoperative adjuvant chemotherapy in rectal cancer operated for cure. Cochrane Database Syst. Rev..

[37] Sargent D., Sobrero A., Grothey A., O’Connell M.J., Buyse M., Andre T., Zheng Y., Green E., Labianca R., O’Callaghan C. (2009). Evidence for cure by adjuvant therapy in colon cancer: Observations based on individual patient data from 20,898 patients on 18 randomized trials. J. Clin. Oncol. Off. J. Am. Soc. Clin. Oncol..

[38] (2020). Cancer Care Ontario: Survival Statistics for Colorectal Cancer. https://cancer.ca/en/cancer-information/cancer-types/colorectal/prognosis-and-survival/survival-statistics.

[39] Patel S., McClintock C., Booth C., Merchant S., Heneghan C., Bankhead C. (2022). The Variations in Care and Real-world Outcomes in Individuals With Rectal Cancer: Protocol for the Ontario Rectal Cancer Cohort. JMIR Res. Protoc..

[40] Thygesen L.C., Ersbøll A.K. (2014). When the entire population is the sample: Strengths and limitations in register-based epidemiology. Eur. J. Epidemiol..

