# Supplementary material for: Colorectal Cancer in Individuals with Cirrhosis: A Population-Based Study Assessing Practice Patterns, Outcomes, and Predictors of Survival
Source: Curr Oncol. 2023 Oct 30;30(11):9530–41. doi: 10.3390/curroncol30110690 (PMC10670829; doi:10.3390/curroncol30110690)
Supplement: Supplementary file 1 [file curroncol-30-00690-s001.zip › curroncol-2671939-supplementary.pdf]

Supplemental Table S1. Data Sources used, with description of data elements.

**Ontario Health Insurance Plan Physician Claims Database (OHIP)**	**The OHIP Physician Claims Database contains all claims for services and procedures performed by physicians and other healthcare providers covered under OHIP. Diagnoses in claims are based on 3-digit ICD-9 coding. Procedures and services, including descriptions and cost, are based on OHIP's Schedule of Benefits and Fees.**
**Ontario Cancer Registry (OCR)**	The Ontario Cancer Registry is the provincial database of information for all Ontario residents who have been diagnosed with cancer (incidence) or who have died of cancer (mortality). Data are collected from hospitals, regional cancer centres, pathology reports and death certificates, and cover the entire province of Ontario.
**Cancer Activity Level Reporting (ALR)**	The data elements constitute patient level activity within the cancer system focused on radiation and systemic therapy services and outpatient oncology clinic visits. The dataset contains clinical, patient level data.
**Canadian Institute of Health Information - Discharge Abstract Database (CIHI-DAD)**	The CIHI-DAD captures administrative, clinical and demographic information on hospital discharges (including deaths, sign-outs and transfers). Includes demographic, administrative and clinical data for inpatient discharges (including surgery).
**Registered Persons Database (RPDB)**	A listing of all persons insured under the Ontario Health Insurance Plan. The data is used to ensure that individuals in other data sources are identified correctly, and to support analysis by demographic groups and geography.
**Postal Code Conversion Files (PCCF)**	The PCCF links postal codes to geographic identifiers and census-based information (including neighbourhood-based income quintile) defined by Statistics Canada.
**National Ambulatory Care Reporting System (NACRS)**	The NACRS contains patient-level administrative, diagnostic, and procedural information for all ambulatory care and emergency department visits.
**Same Day Surgery Database (SDS)**	The SDS contains patient-level administrative, diagnostic and procedural information for all day surgery and outpatient clinic visits.
**Ontario Laboratory Information System (OLIS)**	The OLIS contains data on pathology testing from a proportion of inpatient and outpatient laboratories in Ontario.
**Public Health Ontario Laboratories Information System (PHOL)**	The PHOL contains data on clinical laboratory testing for numerous disease pathogen.

Supplemental Table S2. Data source and data element codes used for cirrhosis, cancer and surgery variables.

Cirrhosis	Data Source	Codes
Cirrhosis of the liver	OHIP	ICD-9 = 571
	CIHI DAD	ICD-9 = 571.2, 571.5<br>ICD-10 = K70.3, K71.7, K74.5, K74.6
Non-bleeding varices	CIHI DAD	ICD-9 = 456.1<br>ICD-10 = I85.9, I98.2, I98.21
**Colorectal cancer**	**Data Source**	**ICD-O-3 Topography**
Colon cancer	OCR	C18, C19
Rectal cancer	OCR	C20
**Colorectal cancer surgery**	**Data Source**	**OHIP Fee Code**
Resection with anastomosis	OHIP	S166, S167, S171, S169, S218
Resection with anastomosis and proximal diversion	OHIP	• (S166, S167, S169, S171) + (S149)<br>• (S213) + (S149, S157)<br>• (S214, S215, S216) + (S149)
Resection without anastomosis (permanent ostomy)	OHIP	S188, S168, S170, S172, S173, S174, S213, S214, S215, S216, S217

Supplemental Table S3. Ontario Health Insurance Plan (OHIP) fee code used to identify diagnostic imaging and referrals.

	OHIP Fee Code
**Chest Imaging**	
CT chest	X406, X407, X125
Chest X-ray	X090, X091, X092
**Abdominal Imaging**	
CT abdomen	X409, X410, X126
MRI abdomen	X451, X455
Abdominal ultrasound	J135, J128
**Colon Assessment**	
CT colonography	X234
Colonoscopy	Z496, Z498, Z494, Z491, Z495, Z555, Z497, Z499, Z492, Z493
Flexible sigmoidoscopy	Z580
**Local Regional Staging**	
MRI pelvis	X461, X465
Transrectal ultrasound	E800 (must be billed with an endoscopic procedure code such as flexible sigmoidoscopy or colonoscopy), J138, J161, J162
**Pre-operative Referrals**	
Medical oncology	A445, A845, A446, A443, A444, A441, A448, C445, C845, C446, C443, C441
Radiation oncology	A345, A745, A346, A343, A344, A341, A348, C345, C745, C346, C343, C341
